# α- and β-Phase Ni-Mg Hydroxide for High Performance Hybrid Supercapacitors

**DOI:** 10.3390/nano9121686

**Published:** 2019-11-25

**Authors:** Jingzhou Yin, Guolang Zhou, Xiaoliang Gao, Jiaqi Chen, Lili Zhang, Jiaying Xu, Pusu Zhao, Feng Gao

**Affiliations:** 1 Jiangsu Key Laboratory for the Chemistry of Low-Dimensional Materials, School of Chemistry and Chemical Engineering, Huaiyin Normal University, Huai’an 223001, China; jiangdazgl@foxmail.com (G.Z.); xlgao15@126.com (X.G.); jqchenhytc@126.com (J.C.); zhaopusu@163.com (P.Z.); 2 State Key Laboratory of Coordination Chemistry, Department of Materials Science and Engineering, Nanjing University, Nanjing 210093, China; xujiaying-1984@163.com; 3 School of Chemistry and Chemical Engineering, Yancheng Institute of Technology, Yancheng 224051, China

**Keywords:** Ni-Mg hydroxides, α- and β-phases, energy storage, supercapacitor

## Abstract

Mg-substituted α- and β-phase nickel hydroxides with high specific capacitance and good stability have been synthesized via sacrificial metal-based replacement reaction. 2D α- and β-phase nickel-magnesium hydroxide (NiMg-OH) have been synthesized by sacrificing magnesium (Mg) powder with nickel salt aqueous solutions. Interestingly, the phase of the obtained NiMg-OH can be controlled by adjusting the nickel precursor. As well, the Mg powder is used not only as Mg source but also alkali source to form NiMg-OH. The α-phase nickel-magnesium hydroxide sample (α-NiMg-OH) exhibits lager surface area of 290.88 m^2^ g^–1^. The electrochemical performances show that the α-NiMg-OH presented a superior specific capacitance of 2602 F g^–1^ (1 A g^–1^) and β-phase nickel-magnesium hydroxide sample (β-NiMg-OH) exhibits better stability with 87% retention after 1000 cycles at 10 A g^–1^. The hybrid supercapacitor composed of α-NiMg-OH and activated carbon (AC) display high storage performance and cycle stability, it presents 89.7 F g^–1^ (1 A g^–1^) and of 0–1.6 V potential window and it maintains capacitance retention of 84.6% subsequent to 4000 cycles.

## 1. Introduction

A supercapacitor is an attractive rechargeable power device for its high-power density, ultrafast charge-discharge performance and prolonged cycling stability [1,2,3,4,5,6,7]. Supercapacitors store energy by means of fast surface redox reactions (pseudocapacitors) or ion adsorption (electric double layer capacitors, EDLCs). An EDLC containing carbon-based electrodes offer excellent power density and cycling life, whereas pseudocapacitors composed of transition metals and polymers deliver high energy density with good cycling stability and power density [8,9,10,11,12,13,14].

Nickel hydroxide (Ni(OH)_2_) is one of interesting electrode substances for high-performance supercapacitors owing to its low cost, high specific capacitance and environmental compatibility [15,16,17]. The α- and β-phases are the common phases of polymorphic forms of layered Ni(OH)_2_ [18]. β-phase Ni(OH)_2_ is the thermodynamically stable phase and commercially applied in alkaline secondary batteries [19]. α-Ni(OH)_2_ is metastable phase but displays a higher theoretical capacitance than that of β-Ni(OH)_2_. However, α-Ni(OH)_2_ tends to convert to β-Ni(OH)_2_ in alkaline solution or when subjected to charge-discharge cycles [20]. Doping or partially substituting with other metal ions, such as Mg [21,22], Al [23,24,25,26], Mn [27], Fe [4], Co [28,29,30,31,32] or Zn [33] in α-Ni(OH)_2_ has found to be an effective way to stabilize the crystal structure; the resulting complex nickel hydroxides have demonstrated much improved electrochemical properties and performance when used as electrodes in supercapacitors. For example, α-phase NiCoMn hydroxide demonstrated high power densities and high energy [34], CoAl hydroxide possessed enhanced electrochemical performance [35,36]. Among these metal ions, Mg is an alkaline-earth metals with smaller atomic weight. Meanwhile, Mg(OH)_2_ is stable in alkaline electrolyte. The brucite Mg(OH)_2_ is isostructural to β-Ni(OH)_2_ with hexagonal scalenohedral symmetry [37]. Guo et al., reported α-phase MgNi hybrid on Ni foam has demonstrated outstanding cycling stability as electrodes for asymmetric supercapacitor [21].

Displacement reaction is an important reaction in inorganic chemistry. Many groups employed and developed the reaction for synthesizing metals or alloys nanostructures [38]. Yet, very little research has addressed the synthesis of metal hydroxides or double metal hydroxides based on the reaction of galvanic replacement reaction. Mg is an active alkaline-earth metals with smaller atomic weight. It can gently react with water to form hydroxyl ion. As well, the solubility of the magnesium hydroxide relatively high in water. The Mg-doped nickel or cobalt hydroxide could be easily obtained when Ni^2+^ or Co^2+^ ions are added to the aqueous solution.

In the present study, both α- and β-phase nanostructured NiMg hydroxides are synthesized at room temperature through reacting Mg powder with a nickel salt aqueous solution. The crystalline phase of NiMg-OH was readily controlled by using different nickel salts as precursors. The crystal and microstructures and electrochemical properties of NiMg hydroxides were systematically characterized and investigated to elaborate the impacts of different crystalline phases on the electrochemical properties of NiMg-OH and the performance of supercapacitors.

## 2. Experimental Section

### 2.1. Preparation of Samples

**Figure 1:**
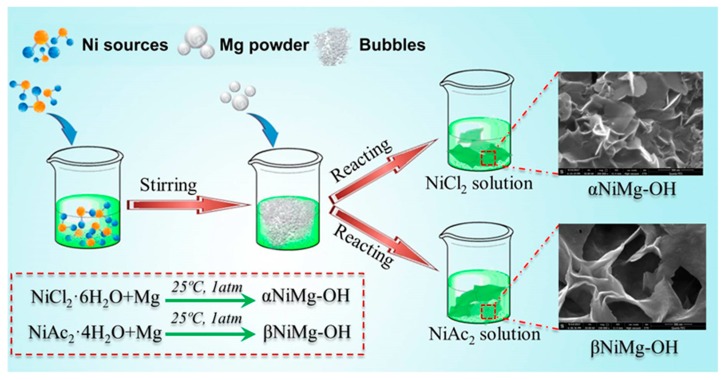
The diagram of the fabrication process for the α-NiMg-OH nanosheets.

The solution-based approach at room temperature is applied to synthesize the different metal hydroxides. Figure 1 displays the diagrammatic sketch of the fabrication process of different phases nickel hydroxides. For synthesis of α-NiMg-OH, 10 mmol of Mg powder was quickly added into the nickel chloride aqueous solution (0.5 M, 200 mL) under stirring. After five minutes of stirring, the mixture was stored in a glass vial at room temperature in ambient atmosphere for 36 h. After the reactions were completed, the apple green precipitates were obtained by centrifugation (4000 rpm, four minutes), rinsed with H_2_O and EtOH, followed by freeze-drying at approximately −45 °C. For synthesis of β-NiMg-OH, the same procedure was used except the NiAc_2_∙6H_2_O as precursor.

### 2.2. Characterization of Samples

The crystalline phases of NiMgOH have been analyzed by X-ray diffraction (XRD) on XRD-6000 (Shimadzu Corporation, Kyoto, Japan). The sample morphology has been studied using scanning electron microscope (SEM, FEI Quanta 450, Hillsboro, OR, USA) and the transmission electron microscope (TEM, JEM-200CX, JEOL, Tokyo, Japan). Brunauer-Emmett-Teller surface areas (BET) were tested via a Micrometrics ASAP 2020 Plus analyzer (N_2_ sorption isotherms, 77 K, Norcross, GA, USA). X-ray photoelectron spectra (XPS) were investigated on a Thermo Scientific Escalab-250xi (Thermo Fisher Scientific, Waltham, MA, USA).The content of Mg^2+^ was detected by inductively coupled plasma optima optical emission spectrometer (ICP-OES) on Optima 2000DV spectrometer (Perkin Elmer Instruments, Shelton, CT, USA).

### 2.3. Electrochemical Measurements

The working electrode was fabricated as follows: The active material (80%, NiMgOH), acetylene black (conductive material, 15%) and polyvinylidene fluoride (PVDF, binder, 5%) were mixed to form slurry by adding a few drops of ethanol. Then, the obtained slurry was coated on pure Ni foam followed by drying at 80 °C for 12 h. All the electrochemical properties were carried out on electrochemical analyzer (CHI 660E, Chenhua, Shanghai, China) in potassium hydroxide solution (KOH, 6 M). The working electrodes have been acquired by compressing the mixture-pasted Ni foam at 10 MPa. The work area of the electrode was 1 × 1 cm^2^ and the mass of the electroactive substance was approximately 2 mg. The three-electrode cell was composed with the nickel hydroxide, Hg/Hg_2_Cl_2_ and platinum. Gravimetric specific capacitance (GSC) has been determined through the galvanotactic charge/discharge (GCD) curve and calculated with *C*_electrode_ *= I*Δ*t/m*Δ*V*, *C* (F g^−1^), Δ*t* (s), *I* (A), where *m* (g) is the specific capacitance, time, discharge current, and mass of the electroactive substance, respectively. Electrochemical impedance spectroscopy (EIS) was tested at open circuit potential (0.01 to 100 kHz) with an AC perturbation of 5 mV.

The asymmetric supercapacitor (ASC) has been assembled with the electrodes of α-NiMg-OH (positive) and active carbon (negative, AC, YP-80F); mass ratio of the active substance in the electrodes follows the equation:

$$m_{+}/m_{-}=C_{-}\Delta V_{-}/C_{+}\Delta V_{+}$$

The analogous electrochemical parameters, such as specific capacitance (*C*_device_, F g^−1^), power density (*P*, W kg^−1^) and energy density (*E*, Wh kg^−1^), have been calculated through the equations [39]:

*C_device_* = *I*Δ*t*/*m*Δ*V*

*P* = 3600*E*/Δ*t*

*E* = 0.5 × *C*Δ*V*^2^/3.6

where *I* (A), Δ*t* (s), *m* (g) and Δ*V* (V) are discharge currents, times, and voltage range, combined mass of the two electroactive substances, respectively.

## 3. Results and Discussion

### 3.1. Characterization Results of NiMg-OH

**Figure 2:**
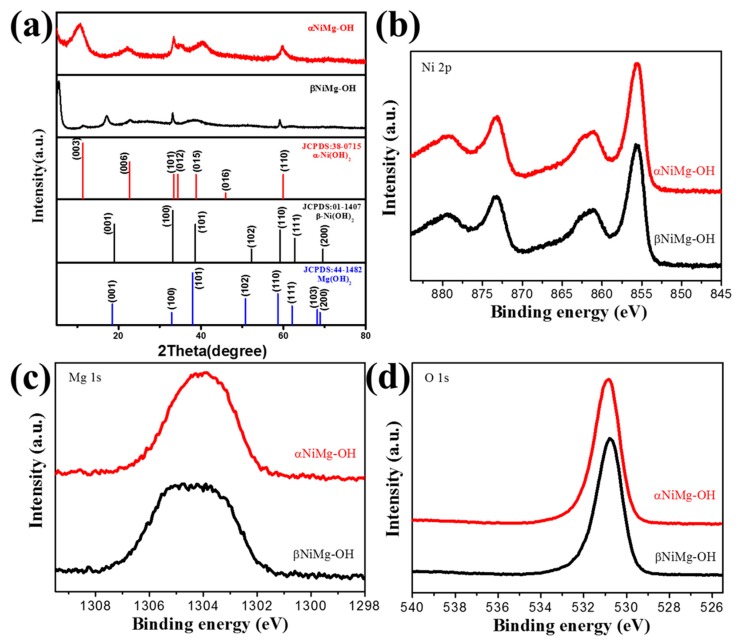
(**a**) XRD patterns of the α-NiMg-OH and β-NiMg-OH, (**b**) Ni 2p, (**c**) Mg 1s and (**d**) O 1s XPS spectra of the α-NiMg-OH and β-NiMg-OH.

The standard cards given at the bottom of Figure 2 were α-phase nickel hydroxide (α-Ni(OH)_2_·0.75H_2_O, JCPDS no. 38-0715), β-phase nickel hydroxide (β-Ni (OH)_2_, JCPDS no. 01-1047) and magnesium hydroxide (Mg (OH)_2_, JCPDS no. 44-1482). For nickel chloride as the nickel precursor (Figure 2a), all diffraction peaks correspond to α-phase nickel hydroxide. The broad diffraction lines of the as-prepared sample indicate the presence of small crystallites. It is noteworthy that there was negative shift for the (003) and (006) planes owing to water molecules and anions in the interlayer space [40] or magnesium doping. The asymmetric peak at 2*θ* = 33.4° signifies the creation of the turbostratic α-phase nickel hydroxide in literatures [41,42]. For nickel acetate as the nickel precursor, the diffraction peaks revealed that the obtained product with mixed phases (Figure 2b). The β-Ni(OH)_2_ structure is isostructural to brucite Mg(OH)_2_. The peaks at 17.2, 33.0, 38.5, 52.1 and 59.0° could be assigned to β-phase nickel hydroxide, with a minor shift for the (001) plane. The remaining peaks at 5.6, 11.3 and 22.5° can be affiliated to the (002), (003) and (006) planes, respectively. This corresponds to the typical α-phase nickel hydroxide structure, except for shifting to lower angles, which resulted from the increased *d*-spacing at the (00*l*) direction through the intercalation of acetate anions or magnesium doping. The similar results were obtained in previous reports [43,44]. The above results show that the anions of the precursors can influence the crystal phase of nickel hydroxide. The XPS results verify the presence of NiMg-OH. Figure 2b–d show the high-resolution XPS spectra for Ni 2p, Mg 1s and O 1s electrons, respectively. Figure 2b display the Ni 2p spectrum can be assigned to the Ni 2p3/2 and Ni 2p1/2 spin orbit levels. The peak at 1204 eV shown in Figure 2c are from Mg 1s electrons [45]. The XPS spectra at 531 eV shown in Figure 2d are from the O1s electrons. The XPS results indicated that the hybrid NiMg-OH were obtained. As well, the mol contents of Mg were further determined by ICP. The content of Mg in the α-NiMg-OH and β-NiMg-OH are 10.4%, 6.6% (mol%), respectively.

**Figure 3:**
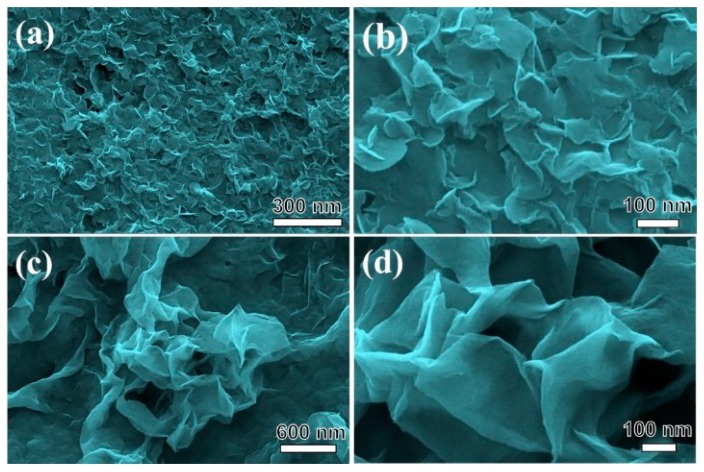
Morphologies (SEM) of the nickel hydroxide with different crystallization phases (**a**,**b**): α-NiMg-OH; (**c**,**d**): β-NiMg-OH.

**Figure 4:**
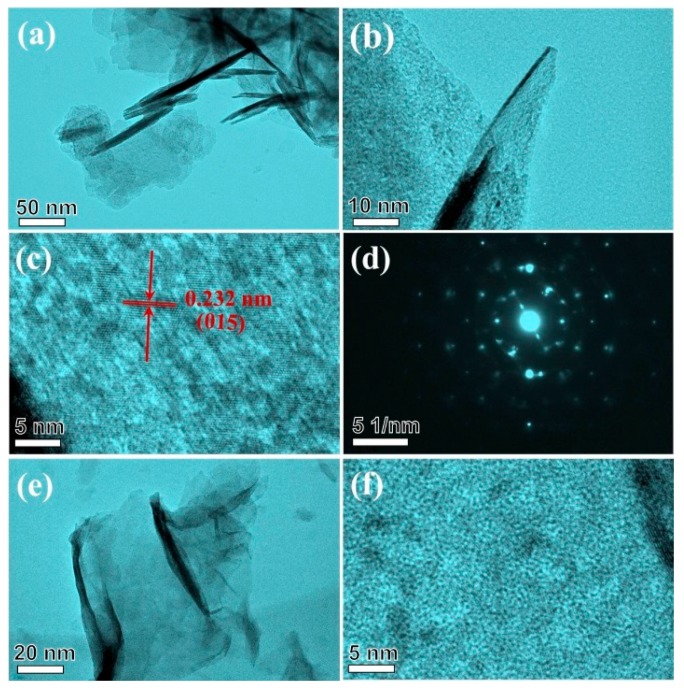
(**a**,**b**) TEMimages; (**c**) High Resolution TEM image; (**d**) SAED pattern of as-obtained products with α-NiMg-OH; TEM image (**e**,**f**) HRTEM image of as-obtained products with β-NiMg-OH.

Morphological features of the different crystalline phase nickel hydroxide were studied by SEM (Figure 3). For α-NiMg-OH obtained from nickel chloride, Figure 3a,b depict nickel hydroxide as a uniform small nanoplate on a large scale with the thickness of <10 nm. For β-NiMg-OH obtained from NiAc_2_, Figure 3c,d depict β-NiMg-OH as a graphene-like 2D nanosheet. As well, the element mapping results showed that Ni and Mg were distributed homogeneous in the two samples (Figures S1 and S2). These results show that the different anions of nickel precursors dramatically influence the growth of nickel hydroxide crystals and the diverse morphologies were further confirmed by TEM images in Figure 4. For α-NiMg-OH, Figure 4a reveals that the sample contains ultra-thin nanoplates. The thickness of a single nanoplate was approximately 1.5 nm (Figure 4b). The TEM images in Figure 4c shows the lattice fringe spacing of 0.232 nm, indicating the (015) plane; diffused circles in the selected area electron diffraction (SAED) pattern (Figure 4d) substantiated the polycrystalline feature of nickel hydroxide nanoflakes. For β-NiMg-OH, the sample has a graphene-like structure with weak crystallization (Figure 4e,f).

**Figure 5:**
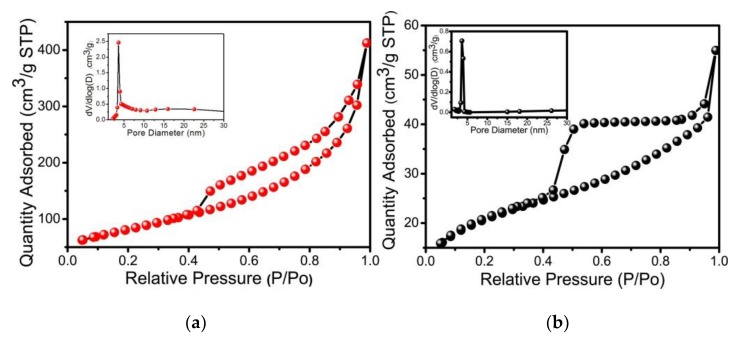
N_2_ adsorption/desorption isotherms and PSD curves (inset) of the nickel hydroxide with different crystallization phases (**a**): α-NiMg-OH; (**b**): β-NiMg-OH.

The BET surface area results were shown in Figure 5 and with pore size distribution (PSD). As presented in Figure 5a,b, the curve type of the nickel hydroxides with different phase can be regarded as type IV (IUPAC classification), indicating the presence of mesopores [46]. It can be noted that for α-phase nickel hydroxide, the isotherm presents a H_3_ hysteresis loop and a greater surface area of 291 m^2^ g^–1^. For β-NiMg-OH, the surface area was 71 m^2^ g^–1^. The PSD of two samples describe a maximum peak centered at approximately 3.6 nm (Table S1). This 3.6-nm mesoporous structure would be beneficial for the unimpeded diffusion of electrode ions into the inner space/matrix of the supercapacitors [47]. This result would be conducive to the outstanding performance of electrochemical tests.

The α- and β-phase NiMg-OH have been prepared by the reaction between Mg powder and a nickel precursor aqueous solution at room temperature. The Mg powder can react in nickel salt aqueous solution with weak acidity for water partly ionizing. The reaction can also be illustrated in the following chemical Equations (1)–(3).

Mg + H_2_O → Mg^2+^ + 2OH^−^ + H_2_↑ (1)

Mg^2+^ + 2OH^−^ → Mg(OH)_2_↓ (2)

Ni^2+^+ 2OH^−^ → Ni(OH)_2_↓ (3)

The reaction process could be divided into two steps: First, the Mg powder reacts spontaneously with hydrogen ion to form Mg ions and hydrogen because the Δ*G* of Equation (1) is −376.4 kJ mol^–1^. Then the hydroxyl ions are surplus in mixed solution. Then, the nickel ions or magnesium ions can combine with hydroxyl ions to form hydroxide. According to the solubility rules if the value of [Mg^2+^] × [OH^–^]^2^ is lower than the value of solubility product equilibrium constant(*K*_sp_(Mg(OH)_2_)), an apple green precipitate of Mg doped nickel hydroxide can obtained [48,49].

In this method, the presence of different anions (chlorine ion and acetate anion) can influence the phase structures (α- or β-phase) of obtained samples. The β-Ni(OH)_2_ structure is isostructural to brucite Mg(OH)_2_ and has hexagonal scalenohedral symmetry [50]. The α-Ni(OH)_2_ has trigonal symmetry containing of planes of β-Ni(OH)_2_ intercalated with H_2_O [37]. For synthesis of the well crystalline β-NiMg-OH, a distorted octahedral coordination complex would be generated at the early reaction stage. Then the amorphous coordination complex can be transformed to [Ni_2_O_4_]^4−^ complex with the process of reaction. At the same time, the Mg^2+^ in the aqueous could replace the Ni^2+^ in the lattice. During the reaction process, the acetate and the [Ni_2_O_4_]^4−^ complex are bound together by hydrogen bonds. As well, the acetate may play important roles as a structure-directing agent, and facilitate the anisotropic growth of β-NiMg-OH, with a typical layer structure [51,52]. For synthesis of α-NiMg-OH, the presence of chlorine ion could not play a role of a structure-directing agent. The uniform small α-NiMg-OH nanoplate were obtained.

### 3.2. Electrochemical Performance of 2D NiMg-OH

**Figure 6:**
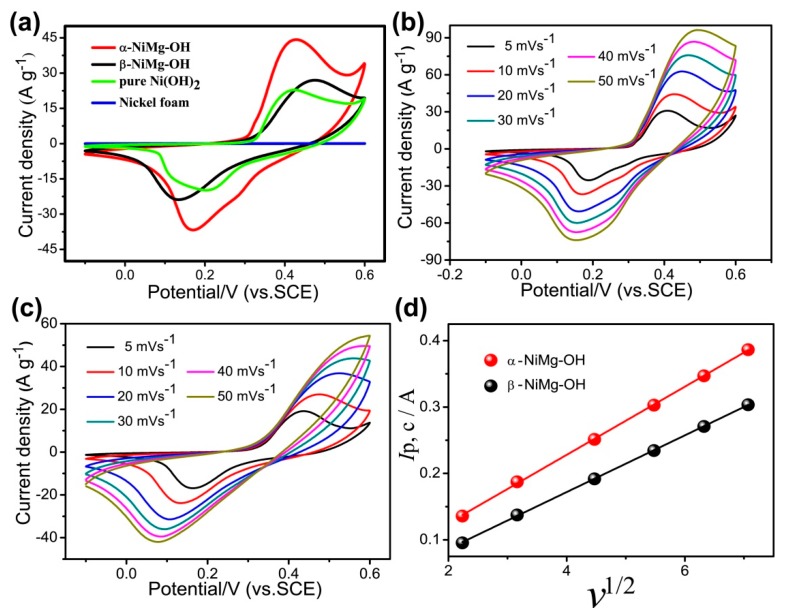
Electrochemical parameters of NiMg-OH and pure Ni(OH)_2_ electrodes in a three-electrode device: (**a**) CV curves at 10 mVs^−1^; (**b**) CV curves of α-NiMg-OH at a range of scan rates; (**c**) CV curves of β-NiMg-OH at a range of scan rates; (**d**) the cathodic peak current versus square root of the scan rate.

Electrochemical performance of nickel hydroxides with different phase has been initially explored by means of the three-electrode configuration in 6 M aq. KOH. Figure 6a depicts that the cyclic voltammogram (CV) curves of α-NiMg-OH, β-NiMg-OH, pure Ni(OH)_2_ and nickel foam electrolytes with potential ranging from −0.1–0.6 V at 10 mV s^–1^. The distinct redox peaks around 0.43 and 0.17 V of α-NiMg-OH electrolyte, and 0.47 and 0.13 V of β-NiMg-OH electrolyte reveal a characteristic pseudocapacitive behavior attributed to the quasi-reversible reactions of nickel hydroxides. The Δ*E* (the potential deviations between the cathodic and anodic peak) of α-NiMg-OH electrode is 220 mV which is smaller than β-NiMg-OH electrode (Δ*E* = 280 mV), indicates that better reversibility than β-NiMg-OH. The flat line of nickel foam indicates that the negligence of its electrochemical performance. Figure 6b,c depict the CV cures of the two kinds of Mg-doped nickel hydroxide samples within 5–50 mV s^–1^, respectively. With an enhanced scan rate, the reduction and oxidation peaks moved to further negative and positive positions, respectively.

Figure 6d depicts the relationship between *I*_p,c_ (the cathodic peak current) and *v*^1/2^(scan rate) of the two kinds of electrodes. The value of *I*_p,c_ increases linearly with the increasing of *v*^1/2^, confirming that two electrodes were limited OH^–^ diffuse to active sites [27]. The slope of the curve *I*_p,c_ vs. *v*^1/2^ for the α-NiMg-OH electrode is larger than that for the β-NiMg-OH electrode indicating the beneficial effect of higher surface area of the α-NiMg-OH electrode. As well, this conclusion can be further confirmed by GCD tests.

**Figure 7:**
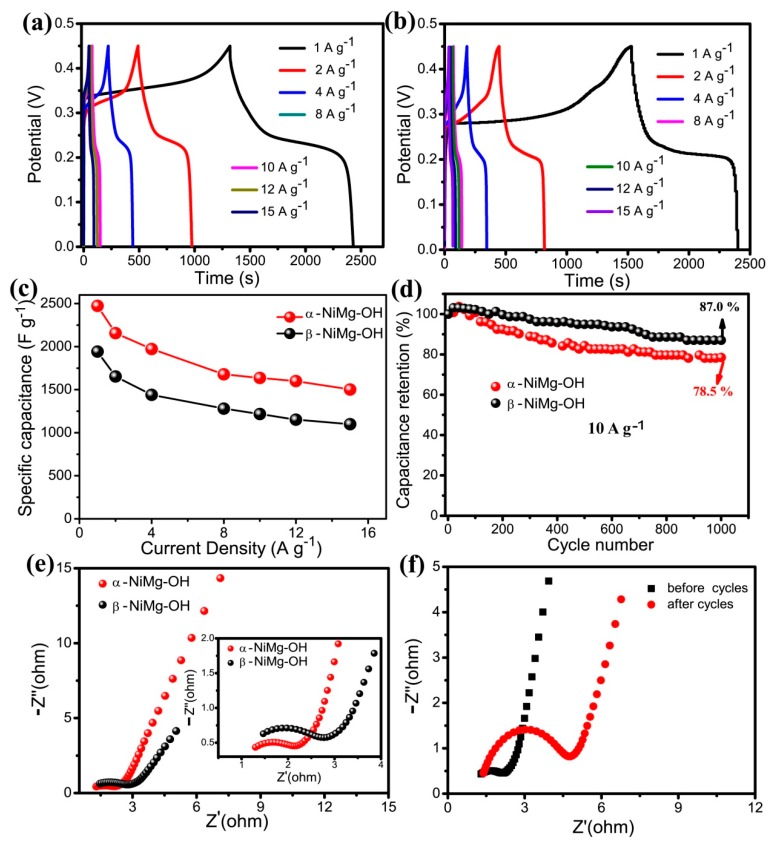
Electrochemical parameters of as-obtained Ni(OH)_2_ electrodes in a three-electrode device: (**a**) GCD curves at a range of current densities of α-NiMg-OH; (**b**) GCD curves at a range of current densities of β-NiMg-OH; (**c**) GSC vs. current density; (**d**) cycling test at 10 A g^−1^; (**e**) EIS spectrum of the two phases Ni(OH)_2_ samples; (**f**) Before and after cycling test of α-NiMg-OH.

The electrode capacitance has been estimated using GCD tests at a range of current densities (Figure 7a,b). The calculated results for α-NiMg-OH (Figure 7c) show that the specific capacitances are 2602, 2156, 1973, 1678, 1636, 1600 and 1502 F g^−1^ at 1, 2, 4, 8, 10, 12 and 15 A g^−1^, respectively. As well, the discharge capacitance at 10 A g^–1^ remained at approximately 70% retention when compared with the capacitance of 1 A g^−1^. Even at 15 A g^-1^, the specific capacitance was approximately 1502 F g^−1^; the result revealed a good charge-storage and rate capabilities. As for β-NiMg-OH, the specific capacitances are 1942, 1653.1, 1441, 1280, 1217.1, 1152 and 1100 F g^−1^ at a current density of 1, 2, 4, 8, 10, 12 and 15 A g^−1^, respectively. The NiMg-OH exhibit superior specific capacitance performance compared other hydroxide materials (Table S2). The cycling stability of the α-NiMg-OH and β-NiMg-OH for practical applications has been evaluated by means of a long-term GCD process at 10 A g^−1^ for 1000 cycles. The β-NiMg-OH exhibits superior cycle stability than α-NiMg-OH with an initial specific capacitance of 87% (Figure 7d). One possible reason is that the delta *G* of the conversion α-Ni(OH)_2_→β-Ni(OH)_2_ is −14.3 kJ mol^–1^ [53], so that α-Ni(OH)_2_ can convert β-Ni(OH)_2_ spontaneously. The α-NiMg-OH maybe convert to β-NiMg-OH with loss capacitance in the cycle process.

These outcomes show that the capacitance of the α-NiMg-OH from NiCl_2_ was better than β-NiMg-OH obtained from NiAc_2_. This property can be further estimated by the EIS results. Figure 7e shows the impedances of the α-NiMg-OH nanosheets and β-NiMg-OH composite electrodes. The EIS curve of α-NiMg-OH has a tiny semicircle in the high frequency region compared with β-NiMg-OH demonstrating lower charge transfer resistance of α-NiMg-OH. The results are consistent with the BET test; the larger electroactive surface can lower the charge-transfer resistance and improve the kinetics. After the cycle test, the larger semicircle diameter demonstrating the charge transfer resistance increases after cycling result in lower the specific capacitance (Figure 7f).

To elucidate the enhanced performance of NiMg-OH with different phase synthesized via this unique method, the Mg doped Ni(OH)_2_ with different content were fabricated by precipitated method is employed as comparison. The specific capacitance at different current densities of Ni(OH)_2_ with different Mg mol content and specific preparation process were shown in Figure S2. The specific capacitance of all Ni(OH)_2_ were less than the NiMg-OH synthesized by sacrificing Mg powder. On the other hand, the specific capacitance of Ni(OH)_2_ improved when the molar ratio of Mg is less than or equal to 10%. However, the specific capacitance of Ni(OH)_2_ reduces when the molar ratio of Mg is 50%. The results show that the appropriate amount of Mg doping can enhance the specific capacitance of Ni(OH)_2_. One possible reason is that the appropriate amount of stable Mg(OH)_2_ in alkaline electrolyte could increase the specific surface area, interlayer distance and pore volume [22]. The ICP results show that the two phase NiMg-OH synthesized via this unique method are 10.35% (α-NiMg-OH) and 6.63% (β-NiMg-OH). The Mg content is useful to improve the electrochemical properties.

### 3.3. Asymmetric Supercapacitor

With the aim of evaluating the practical utility of the fabricated nanosheets, an ASC has been fabricated with α-NiMg-OH nanosheets and AC YP-80F. The capacitive performance of YP-80F was investigated before measuring the performance of the ASC. Figure S3 shows the CVs of YP-80F under a range of scan rates from 0 V to −1.0 V. The quasi-rectangular CV curves indicate the electrical double-layer capacitance phenomenon of the YP-80F electrode. The GCD curves of YP-80F were shown in Figure S3. The YP-80F achieves specific capacitance of 192.9 and 145.0 F g^−1^ at 1 and 10 A g^−1^, respectively.

**Figure 8:**
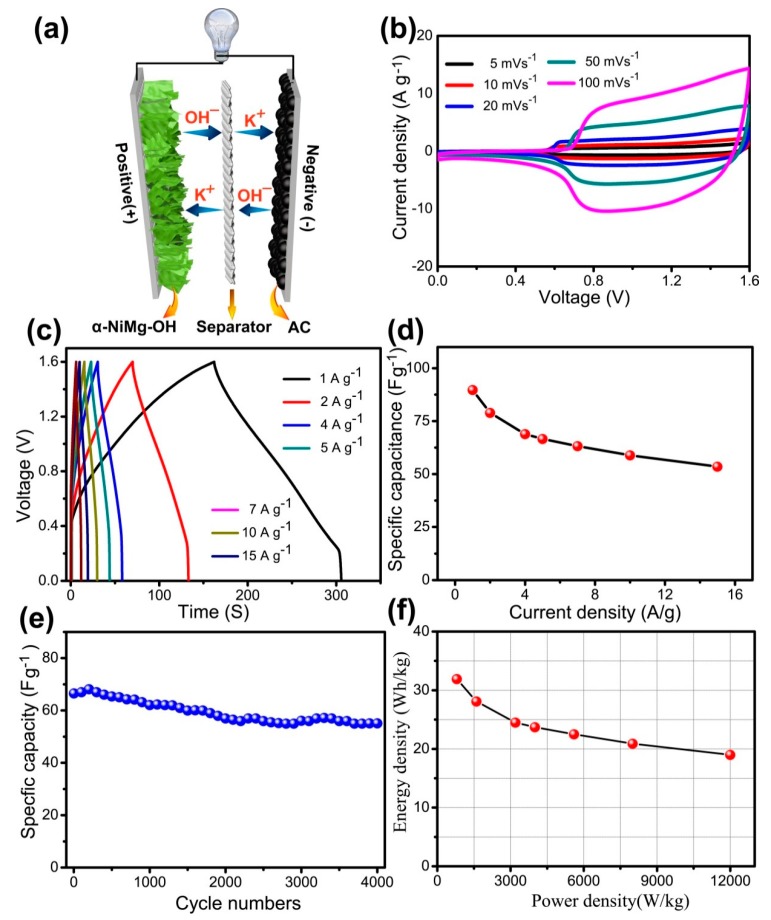
Electrochemical parameters of the two-electrode ASC with α-NiMg-OH nanoplates and AC as the electrodes in 6 M aqueous KOH. (**a**) Schematic illustration of α-NiMg-OH//AC asymmetric supercapacitor configuration; (**b**) CV curves at a range of scan rates with voltages ranging from 0–1.6 V; (**c**) GCD curves at a range of current densities; (**d**) GSC vs. current density; (**e**) cycling stability at 5 A g^−1^; (**f**) Ragone plot of the α-NiMg-OH//AC asymmetric supercapacitor device.

Figure 8a depicts the schematic illustration of a NiMg-OH//AC asymmetric supercapacitor configuration. Figure 8b depicts the CV curves of ASC at a range of scan rates, which lie within 10–200 mV s^−1^ with a potential ranging from of 0–1.6 V. The anodic at 1.1 V and 0.8 V, respectively ascribe to reversible reactions of Ni(OH)_2−x_. GCD curves at a range of current densities are almost symmetric, signifying the occurrence of reversible redox reactions (Figure 8c). These results are consistent with CV measurements. The calculated capacitances are 89.7, 78.9, 68.9, 66.6, 63.2, 58.9 and 53.5 F g^−1^ at 1, 2, 4, 5, 7, 10 and 15 A g^−1^ (Figure 8d). These high capacitances may be ascribed to their distinctive ultra-thin structures. The cycle stabilities of ASC for 4000 cycles at 5 A g^−1^ show that 84.6% of its initial capacitance is maintained (Figure 8e). These outcomes exhibit the good stability of the assembled ASC. The possible reason for the fading capacitance may be due to the increase of ohmic resistance of the ASC after cycling (Figure S5). Figure 8f shows the Ragone plot of ASC.

The energy densities were calculated as 31.9 Wh kg^−1^ at 800 W kg^−1^ and 19 Wh kg^−1^ at 12 kW kg^−1^. These results exhibit the excellent stability of the assembled ASC. Figure S6 shows the schematic illustration of the advantages of α-NiMg-OH nanosheets electrode for supercapacitor properties. The energy density of the ASC was greater than that of Ni(OH)_2_//AC (12.6 Wh kg^−1^ at 1.67 kW kg^−1^) [54], Ni-Al layered double hydroxide//AC nanofibers (20 Wh kg^−1^ at 0.75 kW kg^−1^) [55], and NiCoOH//AC (19.2 Wh kg^−1^ at 80.5 W kg^−1^) [56], and similar to 3D porous Ni(OH)_2_//HPC (34.9 Wh kg^−1^ at 800 W kg^−1^) [11]. The greater electrochemical performance of this device is ascribed to its higher specific capacitance (1171 C g^–1^) and wide potential range (1.6 V).

## 4. Conclusions

In summary, 2D NiMg-OH have been facilely synthesized by sacrificing Mg powder with nickel salt precursors at room temperature. The α- and β-phase of NiMg-OH can be obtained through different nickel salt precursors. This facile and economic process can also be extended for synthesizing other metal hydroxides like cobalt hydroxide. The as-obtained α-NiMg-OH nanosheets displayed high-capacitance i.e., 2602 F g^–1^ (1 A g^–1^), and a retention of 1502 F g^–1^ (15 A g^–1^). In the meantime, approximately 80% capacitance is retained following 1000 cycles at 10 A g^–1^. Importantly, as for ASC, the possessing specific capacitance of 89.7 F g^–1^ of 1 A g^–1^, within 0–1.6 V and a good electrochemical capacitance retention of 84.6% of initial capacitance after 4000 cycles. Moreover, the ASC possessed a gravimetric energy density of 31.9 Wh kg^–1^ at 800 W kg^–1^. Therefore, this work reports a facile process for preparing 2D nickel hydroxides with different phases and also highlights the path for other metal hydroxides.

## Supplementary Materials

The following are available online at https://www.mdpi.com/2079-4991/9/12/1686/s1, Figure S1: SEM and corresponding element mapping of α-NiMg-OH, Figure S2: SEM and corresponding element mapping of β-NiMg-OH, Figure S3: Specific capacitance of Mg doped Ni(OH)_2_ with different content fabricated by precipitated method at different current densities, Figure S4: (a) GCD curves of AC at a range of current densities measured in a three-electrode device; (b) Charge-discharge curve of AC at a range of current densities; (c) Specific capacitance of AC at different current densities, Figure S5: Nyquist plots of electrode α-NiMg-OH before and after charge-discharge cycling. Figure S6: The schematic illustration of the advantages of α-NiMg-OH nanosheets electrode for supercapacitor properties. Table S1: Summary of surface area, Table S2: Comparison between the supercapacitive performances of our work and the recent hydroxide nanomaterials from literatures.
