# Supplementary material for: α- and β-Phase Ni-Mg Hydroxide for High Performance Hybrid Supercapacitors
Source: Nanomaterials (Basel). 2019 Nov 25;9(12):1686. doi: 10.3390/nano9121686 (PMC6955959; doi:10.3390/nano9121686)
Supplement: Supplementary file 1 [file nanomaterials-09-01686-s001.pdf]

Supplementary Materials: α- and β-Phase Ni-Mg Hydroxide for High Performance Hybrid Supercapacitors

Jingzhou Yin [1,2,*], Guolang Zhou [1], Xiaoliang Gao [1], Jiaqi Chen [1], Lili Zhang [1,*], Jiaying Xu [2,3], Pusu Zhao [1] and Feng Gao [2,*]

1. Jiangsu Key Laboratory for the Chemistry of Low-Dimensional Materials, School of Chemistry and Chemical Engineering, Huaiyin Normal University, Huai'an 223001, China; jiangdazgl@foxmail.com (G.Z.); xlgao15@126.com (X.G.); jqchenhytc@126.com (J.C.); zhaopusu@163.com (P.Z.)
2. State Key Laboratory of Coordination Chemistry, Department of Materials Science and Engineering, Nanjing University, Nanjing 210093, China; xujiaying-1984@163.com (J.X.)
3. School of Chemistry and Chemical Engineering, Yancheng Institute of Technology, Yancheng 224051, China

* Correspondence: jingzhouyin@hytc.edu.cn (J.Y.); zll@hytc.edu.cn (L.Z.); fgao@nju.edu.cn (F.G.)

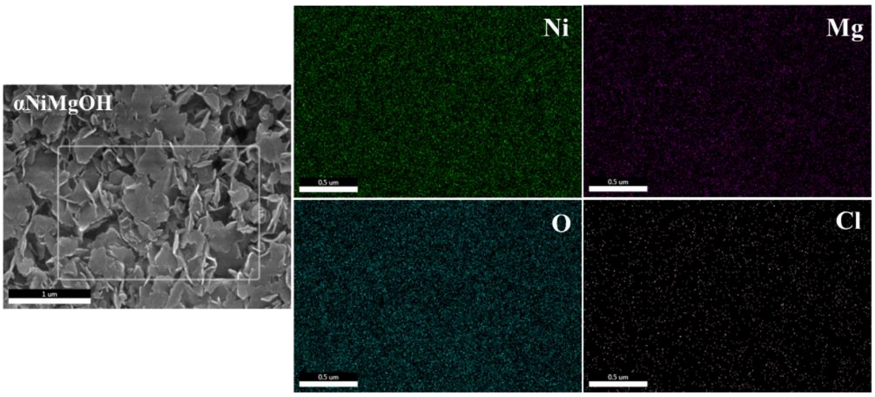

Figure S1. SEM and corresponding element mapping of α-NiMgOH.

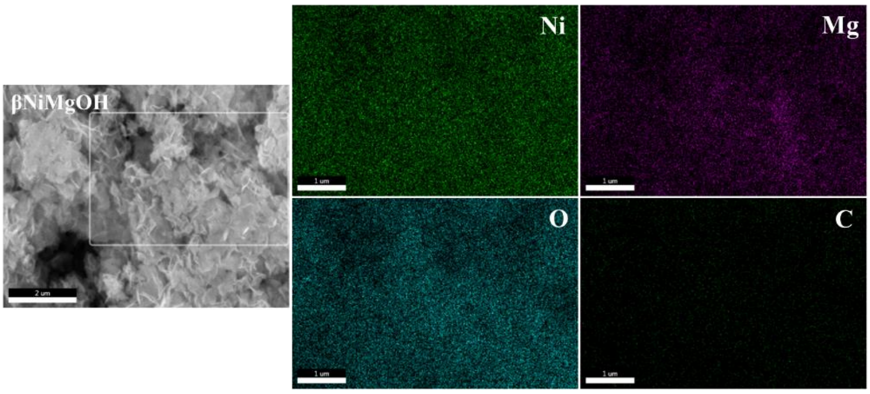

Figure S2. SEM and corresponding element mapping of β-NiMgOH.

Table S1. Summary of surface area

Sample	S_{Total}^{a} (m^2/g)	V_{Total}^{b} (cm^3/g)	S_{Micro}^{c} (m^2/g)	S_{Exter}^{d} (m^2/g)
α-NiMg-OH	290.88	0.0047	12.9	277.98
β-NiMg-OH	71.10	0.0063	12.72	58.39

a: S_{Total} represent BET surface area. **b**: V_{Total} represent t-Plot micropore volume. **c**: S_{Micro} represent t-Plot micropore area. **d**: S_{Exter} represent t-Plot external surface area.

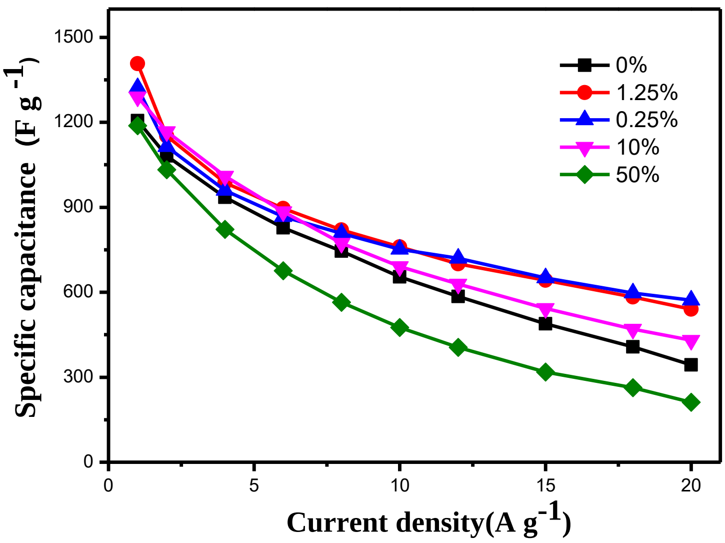

Figure S3. Specific capacitance of Mg doped $Ni(OH)_2$ with different content fabricated by precipitated method at different current densities

Synthesis process: The solution with different Mg content were mixed with $NiCl_2$ aqueous (0.5 mol/L) and $MgCl_2$ aqueous (0.5 mol/L). Then KOH aqueous solution (1.0 mol/L) was added the mixed solution with continuous stirring. The molar ratio of Mg^{2+} + $Ni^{2+}$and OH^- is 1:1. After the reactions were finished, the apple green precipitates were obtained by centrifugation (4,000 rpm, four minutes), rinsed with H_2O and EtOH, followed by freeze-drying at approximately −45°C.

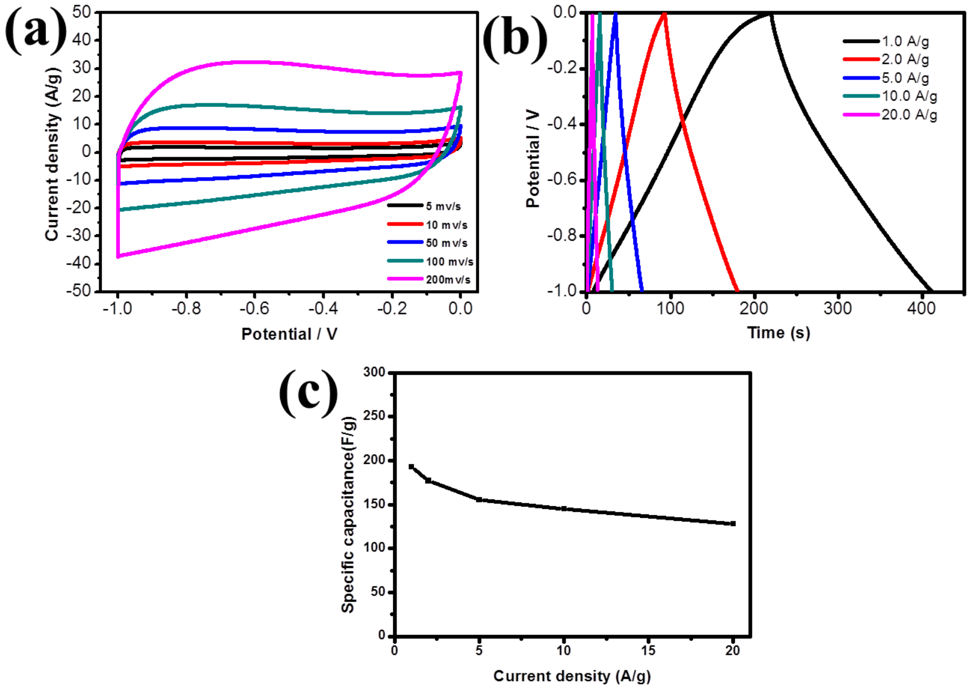

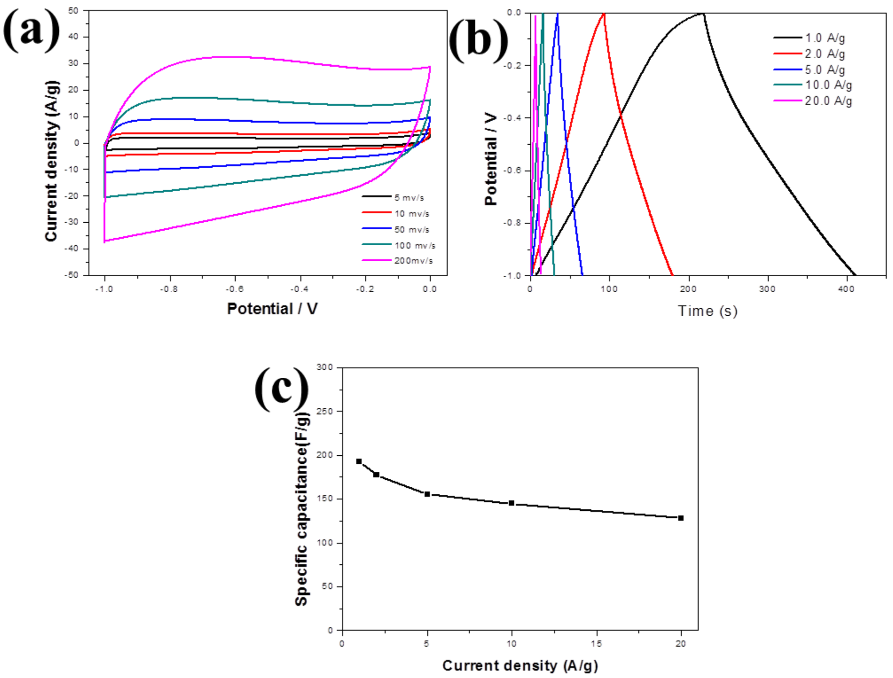

Figure S4. (**a**) GCD curves of AC at a range of current densities measured in a three-electrode device; (**b**) Charge-discharge curve of AC at a range of current densities; (**c**) Specific capacitance of AC at different current densities.

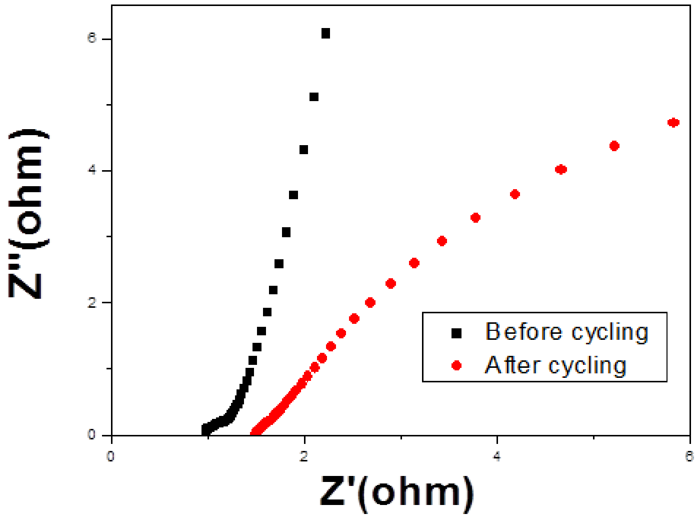

Figure S5. Nyquist plots of electrode α-NiMg-OH before and after charge-discharge cycling.

Table S2. Comparison between the supercapacitive performances of our work and the recent hydroxide nanomaterials from literatures.

**Electrodes**	**Specific Capacitance (F/g)**	**Rate performance**	**Cycling performance**	**Reference**
NiAl-LDH	735 (1 A/g)	75% (1-25 A/g)	116.5% (8 A/g, 1000 cycles)	[1]

NiMgAl-LDH	230.11 (2 A/g)	45.7% (1-3 A/g)	86.1% (5 A/g, 5000 cycles)	[2]
Ni-Al LDH/NNDG	1950 (1 A/g)	75% (1-10 A/g)	95% （10A/g, 10000 cycles）	[3]
NiAl-LDH	2123.7 (0.5 A/g)	50.5% (0.5-20 A/g)	91.9% (5 A/g, 10000 cycles)	[4]
NiMn-LDH/NDCF	2128.3 (0.5 A/g)	70% (0.5-10 A/g)	94.3% (2 A/g, 5000 cycles)	[5]
NiFe-LDH/RGO	1325 (5 A/g)	86.7% (5-20 A/g)	64.7% (15 A/g, 2000 cycles)	[6]
MgAl-LDH/RGO	1334 (1 A/g)	46% (1-10 A/g)	87% (5 A/g, 10000 cycles)	[7]
$Ni(OH)_2$	1567 (1 A/g)	25% (1-10 A/g)	90% (10 A/g, 600 cycles)	[8]
α--$Ni(OH)_2$	1759 (1 A/g)	50% (1-20 A/g)	90.3% (10 A/g, 1000 cycles)	[9]
Mg-$Ni(OH)_2$	1931 (0.5 A/g)	77% (0.5-20 A/g)	95% (10 A/g, 10000 cycles)	[10]
$Ni(OH)_2$	2606 (1 A/g)	44.5% (1-20 A/g)	~20% (10 A/g, 1200 cycles)	[11]
NiMg-OH	2602 (1 A/g)<br>1942 (1 A/g)	~70% (1-10 A/g)	78.5% (10 A/g, 1000 cycles)<br>87% (10 A/g, 1000 cycles)	This work

LDH: Layered Double Hydroxide

NNGO: Nitramine-N-Doped Graphene

NDCF: Nitrogen Doped Carbon Foams

RGO: Reduced Graphene Oxide

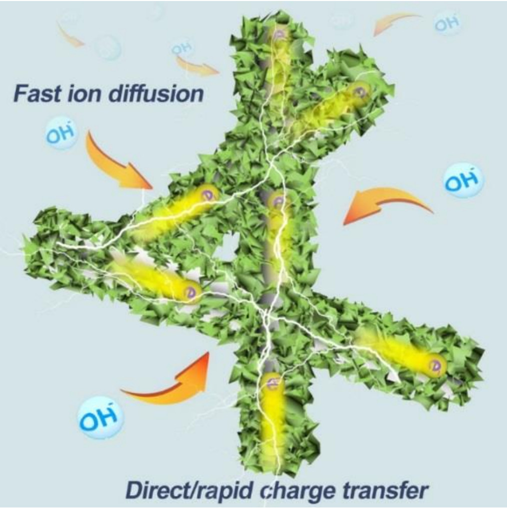

Figure S6. The schematic illustration of the advantages of α-NiMg-OH nanosheets electrode for supercapacitor properties.

References

1. Shao, M.; Ning, F.; Zhao, Y.; Zhao, J.; Wei, M.; Evans, D.G.; Duan, X. Core–shell layered double hydroxide microspheres with tunable interior architecture for supercapacitors. *Chemistry of Materials* **2012**, *24*, 1192-1197.

2. Chuan, J.; Qiang, Z.; Xiaoying, L.; Yuxiang, C.; Xin, W.; Luhao, X.; Hao, Z.; Decai, W.; Wenzheng, Z.; Fan, D., *et al.* Design and fabrication of hydrotalcite-like ternary nimgal layered double hydroxide nanosheets as battery-type electrodes for high-performance supercapacitors. *RSC Advances* **2019**, *9*,

9604-9612.
3. Tian, H.; Bao, W.; Jiang, Y.; Wang, L.; Zhang, L.; Sha, O.; Wu, C.; Gao, F. Fabrication of ni-al ldh/nitramine-n-doped graphene hybrid composites via a novel self-assembly process for hybrid supercapacitors. *Chemical Engineering Journal* **2018**, *354*, 1132-1140.
4. Li, X.; Yu, L.; Wang, G.; Wan, G.; Peng, X.; Wang, K.; Wang, G. Hierarchical nial ldh nanotubes constructed via atomic layer deposition assisted method for high performance supercapacitors. *Electrochimica Acta* **2017**, *255*, 15-22.
5. Chen, D.; Yan, S.; Chen, H.; Yao, L.; Wei, W.; Lin, H.; Han, S. Hierarchical ni–mn layered double hydroxide grown on nitrogen-doped carbon foams as high-performance supercapacitor electrode. *Electrochimica Acta* **2018**, *292*, 374-382.
6. Li, M.; Jijie, R.; Barras, A.; Roussel, P.; Szunerits, S.; Boukherroub, R. Nife layered double hydroxide electrodeposited on ni foam coated with reduced graphene oxide for high-performance supercapacitors. *Electrochimica Acta* **2019**, *302*, 1-9.
7. Hatui, G.; Nayak, G.C.; Udayabhanu, G. One pot solvothermal synthesis of sandwich-like mg al layered double hydroxide anchored reduced graphene oxide: An excellent electrode material for supercapacitor. *Electrochimica Acta* **2016**, *219*, 214-226.
8. Parveen, N.; Cho, M.H. Self-assembled 3d flower-like nickel hydroxide nanostructures and their supercapacitor applications. *Scientific reports* **2016**, *6*, 27318.
9. Li, L.; Xia, G.; Yu, W.; Lu, K.; Zhang, A.; Wang, S. Low-energy hydrothermal fabrication of α-ni(oh)$_2$ nanosheet arrays as efficient electrodes for sustainable supercapacitors. *Sustainable Materials and Technologies* **2019**, *20*, e00085.
10. Xie, M.; Duan, S.; Shen, Y.; Fang, K.; Wang, Y.; Lin, M.; Guo, X. In-situ-grown mg(oh)$_2$-derived hybrid α-ni(oh)$_2$ for highly stable supercapacitor. *ACS Energy Letters* **2016**, *1*, 814-819.
11. Ge, W.; Peng, W.; Encinas, A.; Ruiz, M.F.; Song, S. Preparation and characterization of flowerlike al-doped ni(oh)$_2$ for supercapacitor applications. *Chemical Physics* **2019**, *521*, 55-60.
